# Misalignment of center of foveal avascular zone and center of photoreceptors in eyes with history of retinopathy of prematurity

**DOI:** 10.1038/s41598-024-52407-7

**Published:** 2024-01-23

**Authors:** Ichiro Maruko, Kotaro Irie, Taiji Hasegawa, Manami Takagi, Tomohiro Iida

**Affiliations:** Department of Ophthalmology, Tokyo Women’s Medical University, 8-1 Kawadacho, Shinjuku-Ku, Tokyo, 162-8666 Japan

**Keywords:** Retinopathy of prematurity, Eye abnormalities

## Abstract

To determine the relationship between the center of the foveal avascular zone (FAZ) and the center of the foveal photoreceptors in eyes with a history of retinopathy of prematurity (ROP). To accomplish this, we reviewed the medical records of patients with ROP who were examined at the ROP Clinic of the Tokyo Women's Medical University Hospital. We studied 43 eyes of 23 children with ROP and 67 eyes of 36 control children without any fundus abnormalities. The optical coherence tomography angiographic (OCTA) *en face* images were used to measure the size and location of the foveal avascular zone (FAZ), and cross-sectional OCT images to measure the central retinal thickness (CRT). Our results showed that the size of the FAZ was significantly smaller in the ROP group (0.200 ± 0.142 mm^2^) than in the control group (0.319 ± 0.085 mm^2^; *P* < 0.01). The CRT was significantly thicker in the ROP group (228 ± 30 µm) than in the control group (189 ± 13 µm; *P* < 0.01). The mean length of the foveal bulge was not significantly different between the two groups. The actual distance of the misalignment between the center of the FAZ and the center of the photoreceptors was significantly greater in the ROP group (50.4 ± 29.5 µm) than in the control group (39.6 ± 21.9 µm; P = 0.001). The correlations between the actual distance of misalignment and the size of the FAZ, CRT, and length of the foveal bulge in both groups were not significant. Despite the significant misalignment in eyes with a history of ROP, the center of the foveal photoreceptors was consistently located within the narrow FAZ which indicates that the development of the FAZ and photoreceptor formation are interrelated.

## Introduction

Retinopathy of prematurity (ROP) is a vison-threatening condition which is most often associated with premature birth^1,2^. It is characterized by an abnormal retinal vasculature development, which can then lead to structural and functional changes of the retina. Optical coherence tomography angiography (OCTA) is a non-invasive technique that can obtain *en face* images of the microvasculature of the retina and choroid^3,4^. Cross-sectional OCT images can be obtained simultaneously with this device. Thus, OCTA allows us to simultaneously examine not only the blood flow in the inner retinal layers but also the morphology of the inner and outer layers.

In an earlier study, we analyzed *en face* OCTA and cross-sectional OCT images. We found that the foveal avascular zone (FAZ) in eyes with prior ROP was significantly smaller than that of normal eyes and also that the inner retina was abnormal^5^. However, the height of the foveal bulge at the center of the foveal photoreceptors in the outer retina was not significantly different from that of normal eyes.

The results of an earlier OCTA study from our laboratory showed that epiretinal membranes (ERMs) were associated with a shrinkage of the inner retinal layers and a constriction of the FAZ^6^. We also found that the center of the FAZ was shifted and not aligned with the center of the foveal photoreceptors^7^. However, the relationship between the center of the FAZ and the center of the photoreceptors has not been determined for eyes with a prior ROP.

Therefore, the purpose of this study was to determine the size and location of the FAZ in eyes with a history of ROP, and to determine the alignment of the center of the FAZ relative to that of the center of the foveal photoreceptors.

## Results

**Figure 1:**
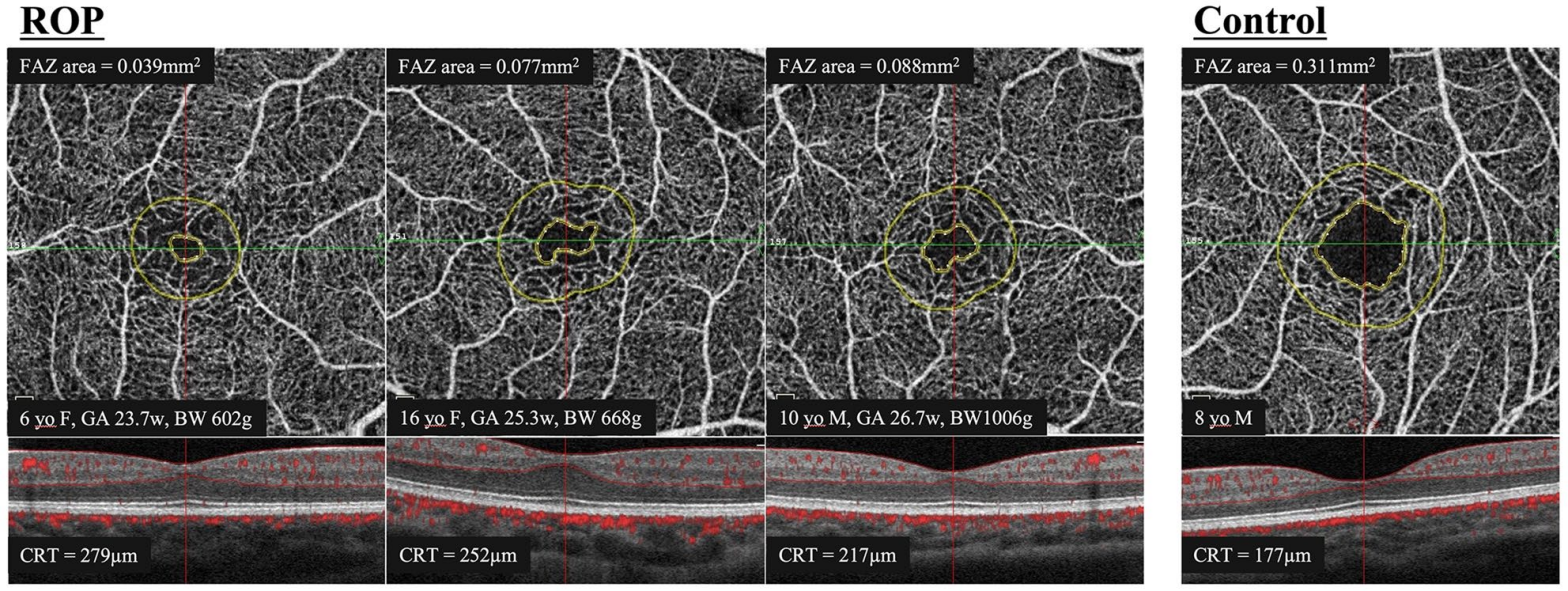
Superficial segmentation of the OCTA images with projected slice navigator aligned to the foveal bulge where the outer segments of the photoreceptor cells is longest in the central fovea. Three cases with a history of the ROP and one case of the control group are presented as representative examples. The center of the foveal bulge was confirmed to be within the FAZ (inner yellow ring) in all cases. The limited outer yellow ring is a 300-µm wide area for the flow density measurement. This yellow line is assigned at the same time when the FAZ is automatically determined by the embedded AngioAnalytics software and is not directly related to this study. *OCTA* optical coherence tomography angiography, *ROP* retinopathy of prematurity, *FAZ* foveal avascular zone, *CRT* central retinal thickness, *GA* gestational age, *BW* birth weight.

We studied 43 eyes of 23 children with a history of ROP whose mean age was 8.7 years with a range of 4 to 16 years. The mean refractive error (spherical equivalent) was -0.22 diopters (D). Five eyes were excluded because the FAZ was too small to examine. For control, we studied 67 eyes of 36 normal children without any fundus abnormalities whose mean age was 10.5 years with a range of 3 to 17 years. The mean refractive error for this group was + 0.22 D. In the ROP group, the mean gestational age was 27.8 weeks with a range of 23.3 to 34.0 weeks, and the mean birth weight was 954 g with a range of 602 to 2035 g. The gestational age in the control group was ≥ 37 weeks which was obtained from the mothers’ medical records. However, there was no detailed birth weight recorded for this control group. All inborn preterm infants in the ROP group underwent ROP examinations at the neonatal intensive care unit of the Tokyo Women’s Medical University and were diagnosed according to the International Classification of Retinopathy of Prematurity^8^. None of the children was diagnosed with Stage 4 or 5 ROP. There were five children with a history of unilateral laser therapy and two children without laser therapy in both eyes; the other 16 children had laser treatment in both eyes. OCTA images of representative cases of the children with a history of ROP and normal children are shown in Fig. 1.

The mean size of the FAZ in the ROP group was 0.200 ± 0.142 mm^2^ which was significantly smaller than the 0.319 ± 0.085 mm^2^ in the control group (*P* < 0.01). The mean central retinal thickness (CRT) was significantly thicker in the ROP group at 228 ± 30 μm than the 189 ± 13 μm in the control group (*P* < 0.01). The mean length of the foveal bulge was 56 ± 12 µm in the ROP group which was not significantly different from the 54 ± 12 µm in the control group (*P* = 0.68: Table 1). The misalignment of the center of foveal photoreceptors and the center of FAZ was 26.9 ± 22.6 µm horizontally and 36.4 ± 29.3 µm vertically in the ROP group and 18.2 ± 17.1 µm horizontally and 27.4 ± 21.6 µm vertically in the control group when analyzed along the X- and Y-axes.

**Table 1:** Mean FAZ area, CRT, and length of foveal bulge in ROP and control groups.

Mean	ROP	Control	P-value
FAZ area (mm^2^)	0.200 ± 0.142	0.319 ± 0.085	< 0.01
CRT (μm)	228 ± 30	189 ± 13	< 0.01
Length of foveal bulge (μm)	56 ± 12	54 ± 12	0.68

*FAZ* foveal avascular zone, *CRT* central retinal thickness, *ROP* retinopathy of prematurity.

**Figure 2:**
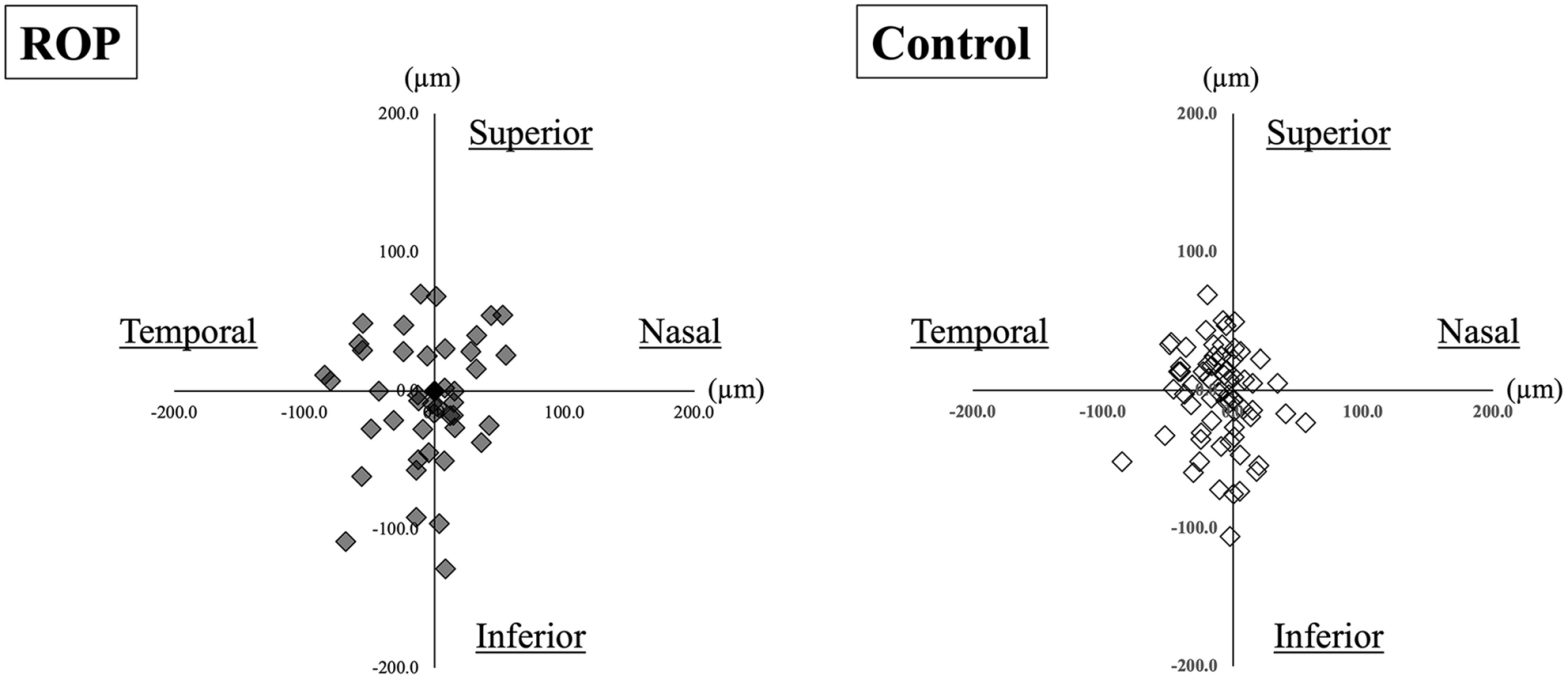
Scatter plot showing the distribution of distance from the center of foveal photoreceptors to the center of FAZ. The distribution of misalignments is determined with the center of foveal photoreceptors as the reference point. The distribution of the misalignments in the ROP and the control groups. The misalignment of the center of FAZ and the center of foveal photoreceptors is 26.9 ± 22.6 µm horizontally and 36.4 ± 29.3 µm vertically in the ROP group and 18.2 ± 17.1 µm and 27.4 ± 21.6 µm vertically in the control group. The differences in the horizontal and vertical distances are not significant between both groups (*P* = 0.06 and *P* = 0.14). *FAZ* foveal avascular zone, *ROP* retinopathy of prematurity.

The distribution of the misalignment of the center of foveal photoreceptors to the center of FAZ is shown in Fig. 2. The misalignment for the ROP group was slightly greater but not significantly than that of the control group for both the horizontal (*P* = 0.06) and vertical (*P* = 0.14) distances. The actual distance of misalignment was 50.4 ± 29.5 µm in the ROP group which was significantly greater than the 39.6 ± 21.9 µm in the control group (*P* = 0.001; Table 2). The center of foveal photoreceptors was confirmed to be within the FAZ in all ROP and control groups (Table 3).

**Table 2:** Misalignment of the center of FAZ and the center of foveal photoreceptors horizontally and vertically.

Misalignment (µm)	X-axes	Y-axes	Actual distance
ROP	26.9 ± 22.6	36.4 ± 29.3	50.4 ± 29.5
Control	18.2 ± 17.1	27.4 ± 21.6	39.6 ± 21.9
P-value	0.06	0.14	< 0.01

Actual distance calculated based on the theorem of three squares.

*FAZ* foveal avascular zone, *ROP* Retinopathy of prematurity.

**Table 3:** Center of foveal photoreceptors was inside or outside the FAZ.

	Photoreceptor center inside FAZ
ROP	43/43 (100%)
Control	67/67 (100%)

*ROP* Retinopathy of prematurity, *FAZ* foveal avascular zone.

**Figure 3:**
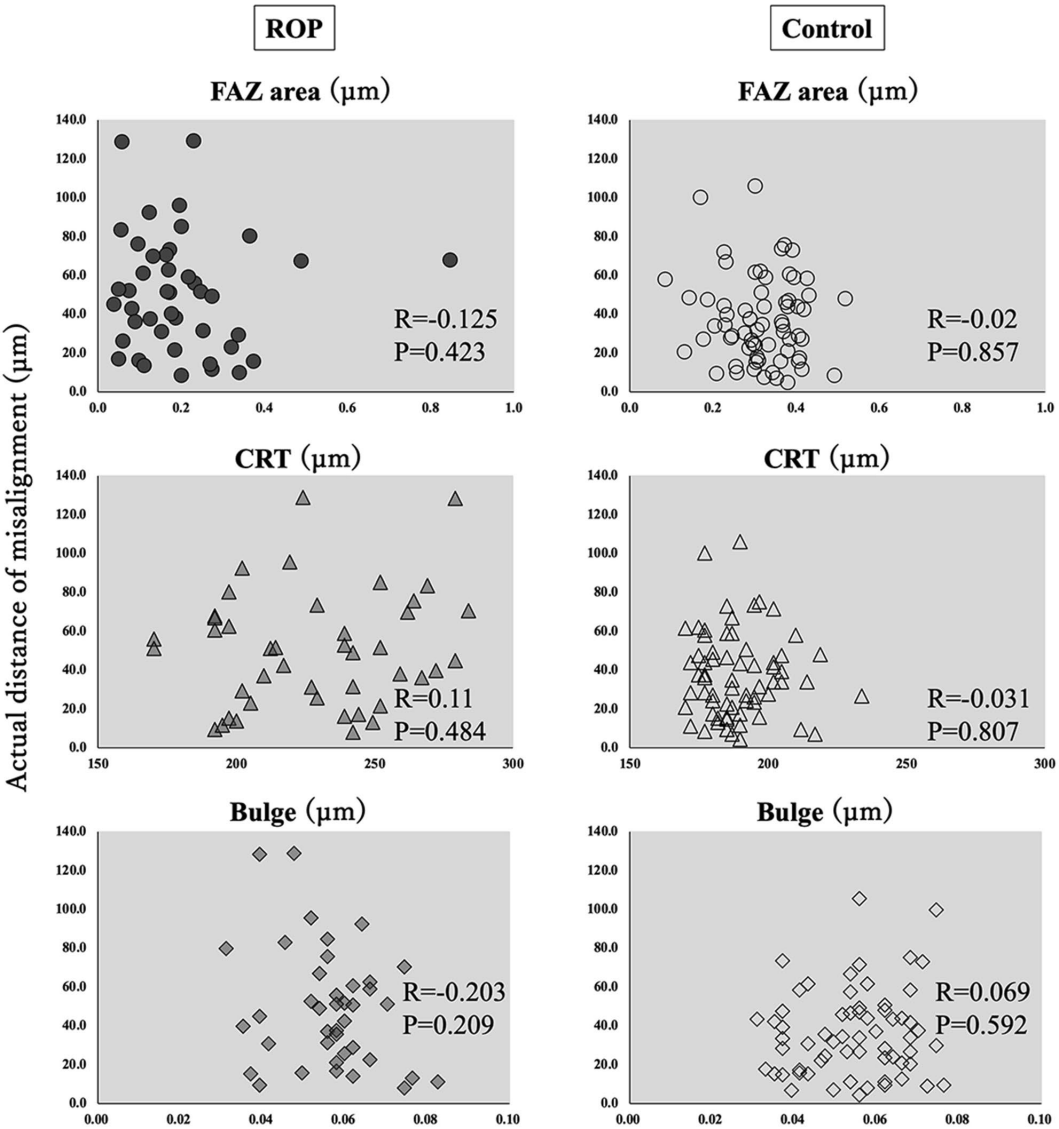
Correlations between the actual distance of misalignment and FAZ area, CRT, and the height of foveal bulge for the ROP and control groups. There were no significant correlations for all comparisons. *FAZ* foveal avascular zone, *CRT* central retinal thickness, *ROP* retinopathy of prematurity.

**Figure 4:**
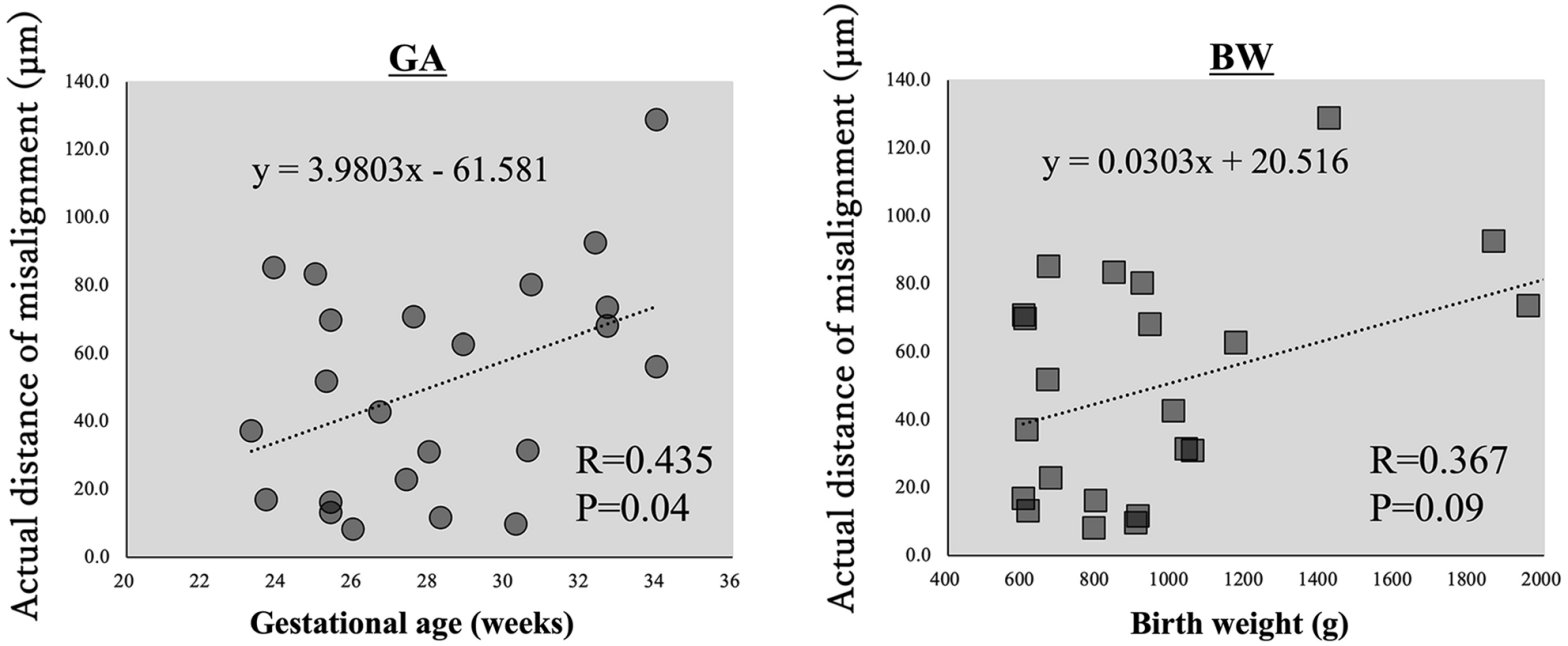
The actual distance of the misalignment of the center of FAZ and the center of foveal photoreceptors was not significantly correlated with the birth weight (R = 0.367, *P* = 0.09), but was positively and significantly correlated with the gestational age (R = 0.435, *P* = 0.04). *FAZ* foveal avascular zone.

The actual distance of misalignment of the center of FAZ and the center of the foveal photoreceptors was not significantly correlated with the size of the FAZ, the CRT, or the length of the foveal bulge in both groups (Fig. 3). The actual distance of misalignment of the center of FAZ and the center of the foveal photoreceptors was not significantly correlated with the birth weight (R = 0.367, *P* = 0.09), but was significantly and positively correlated with the gestational age (R = 0.435, *P* = 0.04; Fig. 4).

## Discussion

Our results showed that eyes with a history of ROP had significantly smaller FAZ which was due to alterations in the inner retinal layer. In addition, the center of the FAZ was not anatomically aligned with the center of the foveal photoreceptors. Nevertheless, the center of the foveal photoreceptors was located within the FAZ in all cases suggesting that the development of the FAZ and photoreceptors may be interrelated regardless of whether the eye is from a normal subject or is associated with an ROP.

A number of significant observations were made regarding the characteristics of the eyes with a prior ROP compared to the eyes of the controls. First, the size of the FAZ was significantly smaller in the ROP group than in the control group as reported^5,9–11^. As reported, the CRT was significantly thicker in the ROP group than in the control group^5,9,11^. This thicker fovea indicates that the formation of the foveal depression is altered which may cause visual dysfunction in ROP patients. Interestingly, no significant difference was found in the mean length of the foveal bulge between the two groups. We conclude that despite the prior ROP, the structural integrity of the outer retina remains essentially unaltered in eyes with good visual acuity at least.

Another interesting finding was the larger misalignment of the center of FAZ and the center of foveal photoreceptors in the ROP group than in the control group. The difference was not statistically significant when analyzed separately along the X and Y axes, but the actual distance of misalignment was significantly greater in the ROP group. This suggested a greater global shift in the overall retinal structure rather than focal changes in the horizontal or vertical axis. The misalignment in the ROP group indicates that this may be another characteristic property of eyes with prior ROP and its characteristics may contribute to a better understanding of the pathologic changes that occur in the eyes of ROP patients. Tunay et al.^12^ reported that fixation was unstable in children with a history of ROP. It is possible that the visual functional abnormalities, such as poor fixation, in a prior ROP may occur even when the visual acuity is good, and it may be related to this misalignment.

It is important to note that the correlations between the actual distance of misalignment and the FAZ size, CRT, and height of the foveal bulge were not significant in both the ROP or control groups. These findings suggested that the cause of the misalignment may not be related to the structural changes in the inner and the outer retina. Interestingly, all of the centers of the foveal photoreceptors that should be highly misaligned in the prior ROP eyes were observed within the FAZ even though the FAZ was smaller in ROP. This suggests that the ROP caused the misalignment of the center of FAZ and the center of foveal photoreceptors, but another mechanism determined the location of the center of the foveal photoreceptors.

A positive and significant correlation was found between the extent of the misalignment and the number of weeks of gestation, but no significant correlation was found with the birth weight. These findings suggested that retinal maturation, as reflected by the gestational age, may influence the foveal development in ROP. However, the positive correlation between misalignment and gestational age in ROP eyes means that the more mature the fovea, the greater the misalignment, which is not consistent with the results in the normal controls. Although there is no clear answer to this question, there may be another mechanism that determines the position of the center of foveal photoreceptors.

The center of the foveal photoreceptors might be located within the FAZ to receive light stimuli without any deflections. Smallman et al.^13^ reported that the photoreceptors can rapidly realign toward the brightest regions of the pupil of the eye after bilateral congenital cataract removal. If a similar phenomenon occurs in the central fovea, it is possible that the center of foveal photoreceptors would be displaced more postnatally, but subsequently shifted into the FAZ area with the development of visual function. Indeed, the formation of the foveal bulge is considered an acquired change that occurs later than the formation of the FAZ^14^.

Many factors influence the development and formation of the inner and outer retinal layers. The FAZ which is related to the vascularization of the inner retinal layer, is smaller in patients with a history of ROP. Congenital macular hypoplasia grade 2 lacks a central foveal depression^15,16^. We have reported that a FAZ cannot be identified in 1.5% of normal eyes by OCTA^17^. This may represent a mild grade 1 macular hypoplasia. As ROP is an acquired disease, evaluations of the FAZ in ROP may provide additional understanding of the development of the fovea.

There are still some important questions regarding the formation of the FAZ that have not been answered. One is whether the central foveal vessels form during embryonic development and are then absorbed, or whether the central foveal vessels are absent from the beginning. Histologically, it was believed that the central foveal vasculature was formed in the early stages of development and then disappeared with the formation of the central foveal depression because ganglion cells were present in the central fovea from the early stages of development^18^. However, subsequent detailed histologic studies have shown that astrocytes that induce vascularity by vascular endothelial growth factor (VEGF), are absent from the fovea in the embryonic stages^19–22^. There would be no astrocytes for retinal vascular growth at the fovea, as if the location of the foveal photoreceptor center is preprogrammed. This suggests that the fovea has a mechanism to inhibit astrocyte entry. The mechanism is still undetermined, but the small FAZ in eyes with a history of ROP suggests that astrocytes enter the fovea from VEGF secretion by the remaining ganglion cells due to premature birth. Given this, the formation of the inner retinal layer is a constant mechanism that cannot be altered.

On the other hand, the morphology of the outer retina including the photoreceptors did not differ from that of the controls in the mean length of the bulge in eyes with a history of ROP. This suggests that the development may be normal regardless of the history of ROP. The fact that the photoreceptor center is located within the FAZ despite the small FAZ may be another indication of normal development. However, because only cases with good visual acuity were examined in this study, this explanation may not apply to all cases. To prove this, it is necessary to investigate the patients immediately after birth which is still technically difficult. The further development of OCT and OCTA that can be performed in the supine position^23,24^ and the mathematical models to simulate retinal angiogenesis may help^25^.

There are limitations in this study. The relatively small sample size can limit the generalization of the findings. In addition, this was a retrospective study and there might have been selection bias.

In conclusion, our results showed that eyes with a history of ROP have a smaller FAZ and the center of the FAZ is anatomically not aligned with the center of the foveal photoreceptors. Nevertheless, the center of foveal photoreceptors is always located within the smaller FAZ. These findings provide significant information in the understanding of the morphologic development of the fovea in eyes with ROP. Further research with larger sample sizes and longitudinal studies are needed to solidify these findings.

## Methods

The procedures used in this retrospective study conformed to the tenets of the Declaration of Helsinki, and they were approved by the Institutional Review Board of Tokyo Women's Medical University. This was a single-center, retrospective, observational, comparative study of patients evaluated at the ROP Clinic of Tokyo Women's Medical University Hospital. All cases had a best-corrected visual acuity (BCVA) of ≥ 20/20 with good fixation. Cases with zone I ROP affecting the foveal vasculature and fixation were excluded. Children with a history of ROP were recruited from the ROP clinic and placed in the ROP group. The full-term infants with normal eyes were recruited from the Strabismus and Amblyopia Clinic of the same hospital and were placed in the control group. All examinations were performed after informed consent was obtained from the patients or parents.

All eyes were examined by OCTA (RTVue AVANTI, Optovue Inc, Fremont, CA) with a scan of 3 × 3 mm centered on the fovea. Cross-sectional OCT images were also obtained with this device. Both eyes were examined, and eyes with poor image quality were excluded. The area of the FAZ (mm^2^) was determined from the *en face* OCTA images and the CRT (µm), which is equal to the thickness of the outer retinal layer, from the cross-sectional OCT images with the embedded software. Patients with a history of ROP were excluded if the FAZ was too small to be measured. The CRT was measured at the peak of the foveal bulge. The foveal bulge was defined as the slight elevation of the ellipsoid zone at the center of the fovea. The highest point of the foveal bulge indicated the center of the photoreceptors, and the length of the foveal bulge, which is equal to the photoreceptor height, was defined between the highest point of the foveal bulge and the retinal pigment epithelium. The slice navigator was aligned with the foveal bulge in the B-scan OCTA image, and the image was projected onto the superficial retinal segmentation using it as the center of foveal photoreceptors and exported to ImageJ (developed by Wayne Rasband, National Institutes of Health, Bethesda, MD; available at rsb.info.nih.gov/ij/index.html), and its X- and Y-coordinates were determined. Also, the area of the FAZ was automatically determined by the embedded AngioAnalytics software. However, there is no embedded program to find the center of the FAZ, so the identified FAZ image was exported to ImageJ. Consequently, the identified FAZ edges and the centroid were manually enclosed with the measurement tool during image analysis. Since it traced the already placed line, the variation was expected to be small. The misalignment of the center of foveal photoreceptors and the center of FAZ in the X- and Y-axis directions was then calculated. The actual distance of misalignment between the center of the foveal photoreceptors and the center of FAZ was also calculated based on the theorem of three squares. The length of the foveal bulge was also measured using ImageJ. The Plot Profile tool in the Analyze menu is used to calculate the distance of the highest intensity between the ellipsoid zone and the retinal pigment epithelium at the center of the fovea. We also determined whether the center of foveal photoreceptors was inside or outside the FAZ. For the control eyes, the coordinate deviation and distance were calculated in the same way for normal children without fundus abnormalities.

### Statistical analyses

All tests to determine the significance of the differences were two-tailed, and a *P* < 0.05 was considered statistically significant. Chi-square tests were used for categorical variables. The means were compared by Mann–Whitney U tests. Spearman’s tests were used to determine the significance of the correlations. All statistical analyses were performed with the EZR free software (Saitama Medical Center, Jichi Medical University, Saitama, Japan), which is a graphic user interface for R (The R Foundation for Statistical Computing, Vienna, Austria)^26^.

## Data Availability

The datasets generated during and/or analyzed during the current study are available from the corresponding author on reasonable request.
